# Precision Aging: Applying Precision Medicine to the Field of Cognitive Aging

**DOI:** 10.3389/fnagi.2019.00128

**Published:** 2019-06-07

**Authors:** Lee Ryan, Meredith Hay, Matt J. Huentelman, Audrey Duarte, Tatjana Rundek, Bonnie Levin, Anja Soldan, Corinne Pettigrew, Matthias R. Mehl, Carol A. Barnes

**Affiliations:** ^1^Department of Psychology, College of Science, University of Arizona, Tucson, AZ, United States; ^2^Department of Physiology, University of Arizona, Tucson, AZ, United States; ^3^Neurobehavioral Research Unit, Division of Neurological Disorders, Translational Genomics Research Institute (TGen), Phoenix, AZ, United States; ^4^Center for Advanced Brain Imaging, School of Psychology, Georgia Institute of Technology, Atlanta, GA, United States; ^5^Clinical and Translational Research Division, Miller School of Medicine, University of Miami, Miami, FL, United States; ^6^Neuropsychology Division, Miller School of Medicine, University of Miami, Miami, FL, United States; ^7^Department of Neurology, School of Medicine, Johns Hopkins University, Baltimore, MD, United States

**Keywords:** aging, cognition, cognitive decline, cognitive impairment, risk for Alzheimer’s disease

## Abstract

The current “one size fits all” approach to our cognitive aging population is not adequate to close the gap between cognitive health span and lifespan. In this review article, we present a novel model for understanding, preventing, and treating age-related cognitive impairment (ARCI) based on concepts borrowed from precision medicine. We will discuss how multiple risk factors can be classified into *risk categories* because of their interrelatedness in real life, the *genetic variants* that increase sensitivity to, or ameliorate, risk for ARCI, and the *brain drivers* or common mechanisms mediating brain aging. Rather than providing a definitive model of risk for ARCI and cognitive decline, the Precision Aging model is meant as a starting point to guide future research. To that end, after briefly discussing key risk categories, genetic risks, and brain drivers, we conclude with a discussion of steps that must be taken to move the field forward.

## Introduction

Cognitive health span does not currently match human lifespan. Sixteen million people in the USA are living with cognitive impairment (Hurd et al., 2013), and more than 1.6 million of these individuals will develop Alzheimer’s disease (AD) annually (Ward et al., 2013). However, it is equally important to note that the majority of older adults—approximately 85%—will not develop AD in their lifetime (Wagster et al., 2012). Nevertheless, many individuals in their 60s and older will experience a range of age-related cognitive impairments (ARCIs) that contribute to decreased quality of life and that have important socioeconomic consequences. ARCI results in three times more hospitalizations—*a $110 billion economic burden to the healthcare system*, loss of independent living—*costing $160 billion in informal and unreimbursed care yearly*, and loss of productivity—*worldwide costs in 2018 are expected to exceed 1 trillion US dollars* (Wimo et al., 2017).

The importance of understanding, preventing, and treating ARCI has resulted in dramatic increases in research over the past 5 years. Most notably, the total National Institute on Aging appropriations doubled from $1.05 B in 2013 to $2.05 B in 2017 (Richard Hodes, Ph.D., Presentation to the NIA Division of Neuroscience Review Panel, October, 2018). While our understanding of the factors that increase risk for ARCI has grown, relatively little progress has been made on how to prevent it. As one example, studies have reported beneficial effects of various exercise programs on cognitive functioning among older adults, both with and without cognitive decline. However, recent reviews have emphasized that the majority of these studies do not find any effect (van Uffelen et al., 2008; Kelly et al., 2014). They note the small number of high quality studies and the large variability in study populations, exercise protocols, and outcome measures as factors that complicate the interpretation of the results. Similar conclusions have been made for studies of cognitive interventions that show small and inconsistent effects (Lampit et al., 2014), although recent studies have suggested that a combination of aerobic exercise and cognitive training may increase the effectiveness of either intervention alone (Karssemeijer et al., 2017; for review see Bamidis et al., 2014).

Without a doubt, ARCI is complicated. Numerous factors increase risk for ARCI including lifestyle choices such as diet, physical activity, and the quality of social interactions, stressors such as chronic illness, bereavement, and depression, peripheral diseases such as heart disease, hypertension, and diabetes, as well as demographic variables that are well known but poorly understood including sex and education. We are also learning about genetic variants that either exacerbate the effects of these myriad risk factors or are protective of them. None of them, however, has provided the magic bullet. Many of these factors have been studied in terms of the impact they have on brain structure and function using neuroimaging methods, or the impact on both general and specific cognitive functions, as well as their impact on risk for disorders of the aging brain, most notably AD. But each risk factor generally explains only some of the variance and often applies to only a subset of individuals. Surprisingly few studies have combined risk factors to understand the degree of variance they explain collectively, and large-scale studies taking an individualized approach to understanding interactions between risks are exceedingly rare. Indeed, we can still say that the overall best predictor of ARCI and risk for AD is, quite simply, age itself.

In this review article, we suggest a reconceptualization of ARCI and our approach to understanding risk, prevention, and intervention. The current “one size fits all” approach to our cognitive aging population is not adequate to close the gap between cognitive health span and lifespan. Here, we present a Precision Aging model, where we apply the concepts that have been developed in the area of precision medicine to understand, prevent, and treat ARCI and cognitive decline. We will discuss how we can reconceptualize multiple risk factors in terms of common categories of risk and their associated pathways or “drivers” of brain function, and how interventions as disparate as exercise and smoking cessation, or tai chi and social interaction, may lead to amelioration of risk for ARCI through similar mechanisms.

## The Promise of Precision Medicine

Precision medicine (National Research Council, 2011) is a broad concept that refers to tailoring therapies to subcategories of disease, based on the specific profile of an individual that is often, but not solely, defined by genomics. A highly successful application of the precision medicine model is in oncology (Vogelstein et al., 2013), where genomic sequencing can be used to classify tumors according to the disordered pathways expressed by a single tumor, rather than classifying tumors based solely on the histological or anatomical tissue of origin. Understanding the underlying mechanisms and factors that encourage tumorigenesis *in a given individual* has led to more precise and effective approaches to treatment.

Precision medicine requires re-evaluation of the way that treatments for disorders are conceptualized and tested in clinical trials (Ashley, 2015). While the comparison between group outcomes in randomized trials may yield statistically significant differences, it is often the case that the mean group difference is driven by a relatively small number of participants who actually respond to treatment. Taking a precision medicine approach, the question is no longer “Does treatment × work?” but “Who does treatment × work for?” Identifying the characteristics of “nonresponders” becomes as important as “responders” in understanding the impact of a particular intervention. Such an approach may result in considerable health benefits by allowing more effective selection of individuals for treatments based on *a priori* known profiles of disease risk and their potential response to treatment.

## The Precision Aging Model: Applying Precision Medicine Concepts to Brain Health and Cognitive Health

Like oncology, the successful application of precision medicine requires a clear goal. We suggest that the goal is to maintain brain health across the full extent of the adult lifespan. That is not to say that age-related changes to the brain can be avoided completely. Some degree of age-related change in brain health and hence, cognitive health, is likely inevitable. In general, however, these cognitive changes are rather subtle and often can be overcome by implementing more efficient strategies for learning, recollecting, and controlling cognition. Many individuals survive into their ’80s and ’90s without debilitating cognitive changes that impair or limit daily functioning. Thus, we define maintenance of brain health and cognitive health as those changes that do not interfere substantially with activities of daily living, and allow continued independence and maintenance of quality of life.

**Figure 1:**
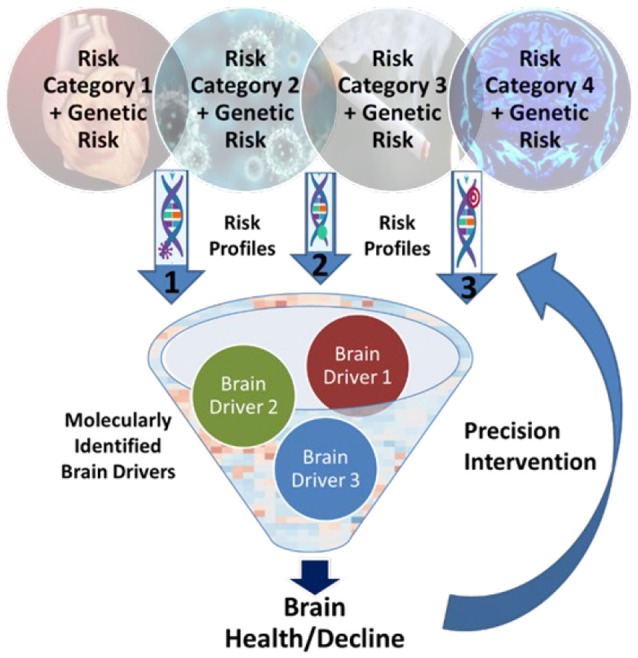
The Precision Aging model. Specific risk factors are grouped into “Risk Categories” that can then be combined with known genetric variants to create individualized profiles of risk for age-related cognitive impairment (ARCI). Understanding the major “Brain Drivers” associated with each category that increase age-related changes in brain structure and function can lead to optimized preventive and therapeutic interventions.

Here, we present a Precision Aging model that reconceptualizes both the risk factors for ARCI and potential targets for prevention and intervention, depicted in Figure 1. First, while there is a long list of known risk factors associated with ARCI, it is often the case that multiple factors can be classified into a single *risk category* because of their interrelatedness in real life. Major risk categories would include, among others, cardiovascular insufficiency, glucose dysregulation, and chronic stress, as well as some that are less well characterized such as immune dysfunction and circadian disruption (see Table 1). Second, all of the individual factors in a risk category likely influence the brain through common *brain drivers*—brain inflammation, compromised brain blood flow, increased neuropathology, and altered synaptic connectivity and function—that exacerbate the aging process and create an environment that is conducive to the accelerated development of neurodegenerative disease. A better understanding of the brain drivers associated with each risk category is key to developing and choosing effective interventions for a given individual. Third, *gene variants* act to either increase the influence of a risk category or protect against it by moderating the impact of brain drivers. Identifying both risk-enhancing and risk-protecting genomic information is critical to understanding the true effect of risk categories on brain health, and becomes more feasible as more individuals have genomic data readily available in their medical records that can be used to support precision genetic profiling.

**Table 1:** Some of the major risk categories for age-related cognitive impairment (ARCI) and cognitive decline, and a list of individual factors that have been associated with each category.

Risk Category	Factors
Cardiovascular Insufficiency	Hypertension, increased body fat/obesity, heart failure, heart disease, high cholesterol, smoking, sedentary lifestyle, poor diet
Glucose Dysregulation	Type 1 and 2 diabetes, prediabetes, poor diet, sedentary lifestyle, increased body fat/obesity, family history
Immune dysfunction	Hormonal changes, environmental exposure to toxins, infections
Chronic Stress	Social isolation, chronic illness, life adversity and loss, trauma, bereavement and grief, caregiving, depression/anxiety, financial hardship
Reserve and Resilience	Educational attainment, early life experiences, occupational complexity, lifelong learning opportunities
Circadian Rhythm Disruption	Sleep disruptions, multi-system dysregulation
Neuropathologies	Plaques, tangles, α synuclein, proteinopathies
Physical Changes	Sensory dysfunction (hearing, vision, balance, olfaction), physical frailty, chronic pain, polypharmacy

*Some risk categories are also commonly related to one another and may share individual risk factors*.

By focusing on commonalities, the Precision Aging model has the potential to both greatly simplify our understanding of risk for ARCI and the impact of a multitude of risk factors on brain structure and function, and provide a way to choose appropriate interventions to mitigate risk. Using this approach, each individual can be characterized based on a profile of risk categories and genetic variants. Importantly, this individualized risk profile can be used to identify targets for prevention of brain injury (for example, increasing physical activity or controlling hypertension) as well as therapeutic interventions to ameliorate the impact of brain drivers already resulting in accelerated age-related brain injury. For this reason, focusing on a single lifestyle or health factor is probably insufficient to reduce risk for ARCI and risk for dementias. The most effective strategy for maintaining brain health will be a combination of approaches—preventative and therapeutic—in order to ameliorate existing brain injury as well as removing, to whatever extent possible, the specific risk factors for a given individual (Baumgart et al., 2015).

Thus, understanding the underlying mechanisms by which risk categories affect brain structure and function is critical for choosing appropriate therapeutic interventions. While some interventions may be “risk specific” (such as grief counseling for the loss of a spouse), others may be more generic (such as mindfulness meditation) and beneficial for multiple specific risks or an entire risk category. Additionally, while various interventions may appear dissimilar on the surface, they, like specific risk factors, may be categorized based on the effect they have on brain drivers. For example, mindfulness meditation, increasing social interaction, and anti-inflammatory medications may all provide the same benefit to the brain by decreasing brain inflammation and oxidative stress.

In the following sections, we unpack each component of the Precision Aging model (Figure 1), including a brief discussion of key known risk categories and some of the specific risk factors that likely cluster together (Table 1), the brain drivers that are the primary mechanisms of brain aging, and how genetic variants may increase risk or provide protection. However, it is important to emphasize that our Precision Aging model is a “work-in-progress.” No doubt other risk categories can be added to our list, and some of the individual constituents within each risk category may be unknown or may be based on limited existing data. There is also a need for a better understanding of how these risk categories relate to brain drivers, as well as the genetic variants that increase sensitivity to, or ameliorate, risk factors within each category. Rather than providing a definitive model of risk for ARCI and cognitive decline, the Precision Aging model is meant as a starting point to guide future research. To that end, after briefly discussing key risk categories, brain drivers, and genetic risks, we conclude with a discussion of steps that can be taken to move the field forward.

## Categories of Risk for Age-Related Cognitive Impairment

In Table 1, individual factors are organized into risk categories based on shared characteristics—they may have a tendency to co-occur in real life, they may share underlying brain drivers, and they may share genetic variants that increase or mitigate risk. Each of the key categories listed could easily warrant a full review that is beyond the scope of current article; instead, we provide a brief overview to highlight the importance of these categories as a starting point in understanding risk for ARCI. Some, such as cardiovascular insufficiency, glucose dysregulation, and chronic stress are well-established. Others, including immune dysfunction and circadian disruption, are relatively novel areas of risk that warrant additional consideration. Others included in the list are reserve and resilience, pathologies, and physical changes.

### Cardiovascular Insufficiency

One of the most visible and best studied risk categories for ARCI and cognitive decline is undoubtedly *cardiovascular insufficiency*, because it is related to many highly prevalent risk factors including hypertension, obesity, hypercholesteremia, heart failure (HF), coronary artery disease, and lifestyle factors including sedentary lifestyle, smoking, and diet. While each factor can occur in isolation, they have a high rate of comorbidity, particularly as severity of any one risk factor increases (Guh et al., 2009). Cardiovascular insufficiency is consistently associated with increased risk for ARCI as well as multiple types of dementias (Corriveau et al., 2016). Most commonly, risk factors in this category have been studied separately, often controlling for other related risk factors. For example, overweight and obese individuals show lower levels of cognitive performance in the areas of executive function, sustained attention, and memory (Cournot et al., 2006; Gunstad et al., 2007; Wolf et al., 2007; Sturman et al., 2008; Volkow et al., 2009) even when controlling for other factors such as hypertension (Walther et al., 2010). Additionally, obese individuals show increased rates of cognitive decline (Elias et al., 2003; Cournot et al., 2006), and increased risk for dementias, including AD (Gustafson et al., 2003; Rosengren et al., 2005; Stewart et al., 2005; Whitmer et al., 2005). Hypertension has been associated with deficits in memory, processing speed, and cognitive flexibility (Hannesdottir et al., 2009; Nguyen et al., 2016) even after optimal medication control (Brady et al., 2005; Verhaaren et al., 2013), as well as increased risk for AD (McGuinness et al., 2009; Power et al., 2018). HF most prominently affects learning and memory, but may also impact information processing speed, attention, language, and executive functions (Mapelli et al., 2011; Miller et al., 2012; Hajduk et al., 2013) and results in faster age-related memory decline (Vogels et al., 2007; Harkness et al., 2011). While cognition can improve following optimal management of HF (Stanek et al., 2009), significant cognitive impairment persists for many individuals, along with a significantly higher risk of dementia compared to age-matched controls (Qiu et al., 2006) Although less common, studies of cardiovascular risk factors in combination with one another provide good evidence that each additional factor adds to an individual’s risk for ARCI (Middleton and Yaffe, 2010; Baumgart et al., 2015; Roberts et al., 2015).

Cardiovascular insufficiency risk factors also share the characteristic that each of them likely contributes to brain aging primarily through chronic systemic and central nervous system (CNS) inflammation and secondarily through the loss of adequate brain perfusion due to vascular damage (Corriveau et al., 2016). Neuroimaging studies consistently find that white matter (WM) appears to be particularly vulnerable to these risk factors. Increases in WM hyperintensity burden on magnetic resonance imaging (MRI) have been associated with hypertension (Raz et al., 2012a,b), obesity (Jagust et al., 2005), and HF (Almeida et al., 2012). WM integrity is negatively impacted by increased body weight, demonstrated using spectroscopy with N-acetylasparate, a microstructural marker of neural viability (Gazdzinski et al., 2008), as well as diffusion tensor imaging (Ryan and Walther, 2014; Kullmann et al., 2016). This association persists even when controlling for other cardiovascular conditions (Bettcher et al., 2013) and hypertension, particularly in frontal cortical regions (Burgmans et al., 2010; Salat et al., 2012).

Cardiovascular insufficiency highlights the complexity of risk for ARCI and the need for longitudinal studies, because some of these relationships may actually change across the lifespan. For example, while there is strong evidence for the link between obesity and increased risk for ARCI, obesity in very late life may be associated with *reduced* risk for ARCI and dementia (Luchsinger et al., 2007; Dahl et al., 2008; Barnes et al., 2009; Fitzpatrick et al., 2009; Gustafson and Luchsinger, 2013) and this may be true for late-life hypertension as well (Kennelly et al., 2009; Corrada et al., 2014).

### Glucose Dysregulation

Closely related to cardiovascular insufficiency is the risk category of glucose dysregulation, including diabetes and prediabetes, a condition characterized by elevated fasting glucose levels, impaired glucose tolerance, and elevated HbA_1c_ levels in the absence of diabetes. Currently, more than 100 million U.S. adults are living with diabetes or prediabetes, according to the National Diabetes Statistics Report (2017) released by the Centers for Disease Control and Prevention. Type 2 diabetes and glucose dysregulation are impacted by both genetics and lifestyle factors such as body composition (e.g., obesity), diet, and physical inactivity. In older adults, diabetes is a risk factor for ARCI, mild cognitive impairment, and dementia (Cukierman et al., 2005; Cheng et al., 2012). Diabetes at midlife is associated with greater ARCI, neurodegeneration, and cerebrovascular disease among older adults (Knopman et al., 2011; Rawlings et al., 2014; Roberts et al., 2014). Especially important is the finding that prediabetes is also associated with greater ARCI (Rawlings et al., 2014). Given that one-third of adults are estimated to have prediabetes (Kalyani et al., 2017), this is an area that clearly warrants further study. Diabetes has been linked to increased global brain atrophy, increased burden of small vessel disease (especially lacunes), alterations in WM, and reductions in functional connectivity among older adults (Biessels and Reijmer, 2014).

### Chronic Stress

Chronic stress is a risk category that has emerged as a consistent predictor of ARCI, cognitive decline, and elevated risk for dementia. Specific risk factors in this category include early and accumulated life adversity (Andel et al., 2011; Korten et al., 2014), chronically experienced social isolation (Cacioppo and Hawkley, 2009; Holwerda et al., 2014), dispositional distress proneness (Wilson R. S. et al., 2005; Wilson et al., 2007; Aggarwal et al., 2014), a life history of depression (Byers and Yaffe, 2011; Cooper et al., 2015), and caregiving for a person with dementia (Allen et al., 2017). Less is known, however, about the brain drivers associated with this category, but these likely include both dysregulated endocrine function (Cacioppo et al., 2015; Lupien et al., 2018) and immune function (Eisenberger and Cole, 2012; Macht and Reagan, 2018), both associated withlow-grade inflammation.

### Physical Changes

Physical changes are well recognized as common correlates of the biological aging process, including sensory changes (loss of vision, audition, vestibular function, olfaction and gustation), frailty (decreases in strength, speed, and balance) and chronic pain. However, the predictive value of these changes for cognitive and health-related outcomes is not well understood (Lara et al., 2015). Physical frailty is characterized by increased vulnerability and depleted physiological reserve associated with age. In particular, frailty disproportionately affects older women, especially African Americans, with older women twice as likely to be frail than older men. Frailty symptoms have been found to be linked to cognition. For example, walking speed and grip strength are associated with reduced executive function and memory (Boyle et al., 2010). In general, as the number of frailty symptoms increase, cognition is poorer (Robertson et al., 2014). Pain is a related and common but understudied area of risk among older adults. It is estimated that 60%–70% of people over 65 years report some degree of persistent pain, and this figure is higher among adults who reside in assisted living and nursing homes. Research indicates that pain is likely to increase with age, be reported more often by women and linked to poor sleep and severity of depression (Molton and Terrill, 2014), but its relationship to cognitive impairment is not well understood.

### Immune Dysfunction

An area that clearly warrants additional investigation is the aging immune system. Despite the importance of inflammation as a putative brain driver associated with many risk factors for ARCI including cardiovascular insufficiency and chronic stress, we know relatively little about the bi-directional immune/inflammatory mechanisms of the brain, how they change with age, and how these age-related changes relate to ARCI and risk for dementia. Inflammation and immunity coexist in the same pathological process and they share the same cellular basis where inflammatory cells are also immune cells (Andreasson et al., 2016). We know that aging substantially affects immune system regulation resulting in defects in both rapid responses to infectious agents (the innate immune system) and the slower generation of antibodies to infectious pathogens (the adaptive immune system; Franceschi et al., 2007; Montecino-Rodriguez et al., 2013; Shaw et al., 2013). The most pronounced changes observed with aging are in the adaptive immune system characterized by a decrease in naive T cells and an increase in memory cells (Hearps et al., 2012). These changes result in a low-grade chronic proinflammatory environment with increased production of proinflammatory cytokines [e.g., IL-6, TNF-α, acute-phase proteins, reactive oxygen species (ROS), and autoantibodies]. Age-related immune system changes are also accelerated by genetic predisposition, hormonal changes with decreased production of estrogens or androgens, mitochondrial function, and metabolic changes in the adipose tissue associated with obesity (Deleidi et al., 2015). Therefore, an individual’s genetic profile, environmental exposures, and infections over a lifetime likely exacerbate the dysregulation of immune responses. However, relatively little is known about the cellular and molecular mechanisms controlling age-related changes in immune function, or the impact they have on risk for ARCI and neurodegenerative disorders (von Bernhardi et al., 2010; Rawji et al., 2016).

### Circadian Disruption

Another emerging area of interest is understanding the mechanisms that drive and coordinate circadian oscillations of multiple organ systems, how they change with age, and how these changes may impact cognition. Circadian disruption is most often studied in the context of physiological arousal and sleep-wake cycles measured by melatonin and cortisol sampling, wearable devices for capturing activity levels, and polysomnography to study neural oscillation characteristics of different sleep stages (for review see Duffy et al., 2015). Importantly, evidence from all methods suggests that increasing age is associated with a disruption in circadian oscillations or rhythms that play an essential role in numerous aspects of health including temperature regulation, hormone release, and sleep (for reviews see Spira et al., 2014; Hood and Amir, 2017).

In mammals, the neurons of the suprachiasmatic nucleus (SCN) in the hypothalamus are the principal drivers of circadian oscillations through synaptic connections with multiple areas including the cerebral cortex, pineal gland, lungs, liver, kidney, heart, and other organs (for review see Hastings et al., 2003). Although the mechanisms underlying age-related circadian disruptions are not fully understood, post-mortem histology has revealed deterioration of SCN neurons in humans (Swaab et al., 1985), which may contribute to circadian disruption in many downstream tissues (for review see Hood and Amir, 2017). While SCN cellular changes are not typically measurable in living humans, several downstream macro-level changes can be observed. For example, earlier phase shifts in body temperature, melatonin, and cortisol rhythms have all been observed in normal aging (Duffy et al., 2015). Melatonin, in turn, promotes sleep onset, which also shifts earlier with age. Age-related sleep disruptions are perhaps the most notable and commonly measured circadian change. Older adults exhibit a strong preference for morningness, more fragmented sleep, and reduced total sleep time compared to younger adults (for reviews see Scullin and Bliwise, 2015; Mander et al., 2017). Critically, emerging longitudinal work from humans suggests that self-reported and objectively measured sleep declines are associated with an increased risk of ARCI and AD, even after accounting for other risk variables including body weight and depression (for review see Spira et al., 2014; Holth et al., 2017; Sterniczuk et al., 2013). However, most research to date has been cross-sectional, and little is known about how age-related disruptions in these rhythms are related to other risk factors and how the interaction between these factors contribute to ARCI within an individual.

### Reserve and Resilience

Finally, in any model of aging, it is important to consider not only those factors that increase risk for ARCI, but also those factors that may protect against it. Reserve and resilience are theoretical concepts suggesting that individual differences in genetic predisposition, combined with lifetime experiences, may modify the brain in a way that allows for the preservation of cognitive health in the presence of age-related brain changes and/or neuropathology (Barulli and Stern, 2013; Reuter-Lorenz and Park, 2014; Stern et al., 2018). These are most commonly measured by socio-behavioral proxies such as educational attainment, IQ, occupational complexity, and cognitively engaging leisure activities. Considerable evidence suggests that these lifetime experiences are associated with a lower risk of ARCI (Roe et al., 2011; Pettigrew et al., 2013) and dementia (for a meta-analysis, see Valenzuela and Sachdev, 2006). Despite the strong evidence for the beneficial effects of these lifetime variables on the risk of developing AD, little is known about their neurobiological basis and how they moderate the impact of the normal aging trajectory and ARCI. Importantly, these socio-behavioral proxies reflect experiences across the lifespan, and may continue to evolve even in mid- to late-life, and as such may provide important avenues for behavioral interventions. The concept of reserve, therefore, provides multiple, possibly time-varying mechanisms by which individual differences in lifetime experiences could influence aging trajectories (Soldan et al., 2017).

## Genetic Influences That Increase Cognitive Risk or Protect Against it

While a detailed discussion of genetically encoded risk and protective factors is beyond the scope of this article, several reviews of this topic are available (Visscher et al., 2012; Hayes, 2013). For most of the risk categories in Table 1, well-powered genome-wide association (GWAS) studies have already been performed. These studies are designed to identify alleles in the population that are statistically enriched or depleted in individuals with the disease (or trait) of interest. The ultimate resulting outcome is a panel of associated alleles that can be utilized to assess each individual’s polygenic risk score (PRS) for the specific risk category. There are published examples of well powered use of the GWAS/PRS approach in cardiovascular insufficiency (hypertension and heart disease), glucose dysregulation (obesity), neuropathological conditions (Alzheimer’s and Parkinson’s), and circadian disruption (sleep phenotypes) among others. For many of the listed risk categories, data exists that would empower an individualized PRS assessment provided that a genome-wide single nucleotide polymorphism profile for the study participant could be generated.

It is important to note that for most, if not all, of the risk categories, a personalized PRS is simply a risk assessment. The risk categories in Table 1 are complex and therefore rely on the interplay between genetic risk, environmental exposure, and lifestyle factors to determine whether an individual will develop disease. For example, an individual may be at high genetic risk for Type II diabetes but may be able to avoid the disease entirely through strict lifestyle choices related to diet and exercise. This example raises important questions that need to be explored more deeply. What if an individual is at high risk for a disorder such as obesity, but is able to avoid obesity entirely? How do their genetic risk factors related to the heightened obesity risk alter their cognitive performance in the absence of disease? In other words, can we observe differential cognitive performance in these individuals in the absence of obesity? An example of this is found in the FTO gene. Specific alleles within FTO are associated with altered risk for obesity (Yang et al., 2012). Interestingly, these same alleles are associated with increased declines in verbal memory among middle-aged adults *in the absence of diabetes, hypertension, and obesity* (Bressler et al., 2013). There are likely many other examples of genetic associations for specific risk factors that exert an influence on cognition even in the absence of disease. This argues strongly for an understanding of an individual’s genetic profile in addition to their “expressed phenotype” (e.g., observable disease), as both measures may contain important information about cognitive health.

## Brain Drivers: Mechanisms of Brain Aging

**Figure 2:**
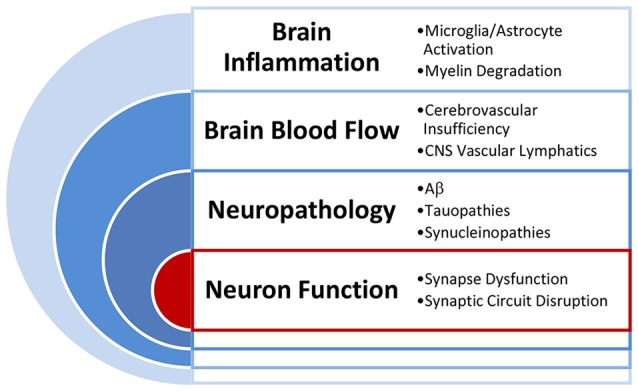
Primary drivers of brain function. Illustration of the interactions of the primary drivers of brain function that work to influence brain health and cognitive outcomes in aging.

The discovery of targets for intervention to promote brain health and to prevent and attenuate ARCI requires identification of drivers of brain and cognitive health that are linked to the major risk categories (Figure 2). Critical categories of drivers that are known to impact brain and cognitive health include brain inflammation (Sankowski et al., 2015; Janota et al., 2016; Clarke et al., 2018; Miners et al., 2018), compromised brain blood flow (Love and Miners, 2016), increased neuropathological markers (Rahimi and Kovacs, 2014) and altered synaptic function and synaptic connectivity (Berchtold et al., 2013). Each of these drivers can be linked to identified risk categories outlined above and can ultimately accumulate and interact to alter neuron function and cognition.

### Brain Inflammation

Inflammation is a physiological function essential for recovery from injury and protection from infection. Under chronic pathological conditions, however, it can activate feed-forward cascades that contribute to neuroinflammation and ultimately neuronal dysfunction and neurodegeneration. This inflammatory cascade is thought to result from cytokine and chemokine activity in endothelial cells of the brain microvasculature as well as within the brain parenchyma affecting microglia, astrocytes, oligodendroglia, and ultimately, neurons.

Brain inflammation is an important driver of brain function and linked to many of the risk categories for ARCI including cardiovascular insufficiency, immune dysfunction, glucose dysregulation, chronic stress and neuropathologies. For example, with regards to cardiovascular risk categories, increases in inflammation are known to occur in normal brain aging (Lynch, 2010; Gabuzda and Yankner, 2013) as well as in systemic inflammatory diseases such as HF, diabetes and hypertension. Studies in HF patients have shown that increases in circulating inflammatory factors such as interleukin-1alpha (IL1α) and interleukin 6 (IL-6) are strongly correlated with decreased cognitive performance (Athilingam et al., 2013; Mann, 2015; Kure et al., 2016). Impairments in memory (Teunissen et al., 2003), executive function (Heringa et al., 2014) and processing speed (Bettcher et al., 2014) as well as general changes in cognitive function (Weaver et al., 2002; Schram et al., 2007) are also associated with circulating increases in cytokines including TNF-α, IL-6, and C-reactive protein.

Overproduction of pro-inflammatory cytokines, including TNF-α, is also a key feature of the pathophysiology of metabolic disorders including type 2 diabetes (for review, see Ferreira et al., 2014). TNF-α is overexpressed in adipose tissue of obese individuals. Interestingly, brain inflammation has been suggested to underlie defective neuronal insulin signaling in individuals with AD (Bomfim et al., 2012). AD and diabetes share common inflammatory signaling pathways, suggesting that mechanisms similar to those that mediate peripheral insulin resistance in type 2 diabetes may also underlie impaired brain insulin signaling and neuronal dysfunction in AD.

Microglia, found in the CNS, are analogous to macrophages in the peripheral nervous system and are the resident immune cells of the brain. Microglia have a pleotropic role in the brain and under normal physiological conditions microglia maintain an M2 phenotype associated with neuroprotection and repair and in the maintenance of healthy synapses *via* synaptic pruning, neurogenesis and immunosurveillance (Hickman et al., 2018). Under chronic neuroinflammatory conditions, however, microglia transition to an M1 activated state which is pro-inflammatory. Activation of microglia has been shown to be linked to the development of neuropathologies which is a major risk category for ARCI. For example, microglia are activated in several neurodegenerative diseases including AD, Parkinson’s disease and multiple sclerosis. Furthermore, microglia can be activated by hypoxia, trauma, stroke and by systemic inflammation such as that observed in patients with HF or diabetes (Nimmerjahn et al., 2005; Durafourt et al., 2012; Melief et al., 2012). Microglia activation results in increased ROS production, cytokine production, and activation of brain inflammatory pathways that contribute to neuronal dysfunction and cognitive impairment, including memory loss (Streit et al., 2004; Hein et al., 2010; Matousek et al., 2010). Recent studies employing epigenetics and transcriptomics have shown that normal aging is associated with regionally-specific changes in glial gene expression patterns (e.g., Soreq et al., 2017).

Activated microglia are also known to result in the up-regulation of astrocyte reactive genes (Liddelow et al., 2017; Clarke et al., 2018) and the conversion of astrocytes from the A2 “neuroprotective” phenotype to the A1 phenotype that induces neuronal and oligodendrocyte cell death. These A1 astrocytes are also linked to the neuropathology risk category for ARCI. During normal aging, astrocytes show region-specific changes in gene expression related to neuroinflammation, with brain areas involved in cognition such as hippocampus and striatum showing greater transcriptional changes than observed in neocortical astrocytes (Clarke et al., 2018).

Oligodendrocytes are responsible for the production and maintenance of myelin in the brain. Myelin is known to show degenerative changes with age (Bennett and Madden, 2014; Lockhart and DeCarli, 2014) that are observable on MRI as increased WM hyperintensities, loss of WM volume both cross-sectionally (Sullivan and Pfefferbaum, 2006) and longitudinally (Resnick et al., 2003) and changes in characteristics of diffusion properties of myelin tracts (Ryan et al., 2011; Salat et al., 2012). How age-related changes in oligodendrocytes are related to changes in neuronal function is an important area of continued study. In addition to providing and maintaining myelin, oligodendrocytes also provide metabolic support for axons. Recent studies have shown that oligodendrocytes are metabolically coupled to their associated axons by providing lactate for the axons to use for ATP production (Fünfschilling et al., 2012). Age-related changes in the metabolic functions of oligodendroglia may further contribute to age-related changes in WM and cognitive function.

In summary, age-related changes in both systemic and central inflammation serve as important drivers of brain health. One would predict that interventions that preserve the health-promoting aspects of glia function, as well as reducing pathophysiological neuroinflammatory cascades, could be particularly effective at attenuating ARCI.

### Brain Blood Flow and Brain Lymphatic Vascular Systems

Adequate brain blood flow is a key brain health driver and directly linked to some ARCI risk factors including cardiovascular insufficiency, glucose dysregulation and immune dysfunction. Maintenance of brain health and homeostasis over the lifespan is dependent on adequate cerebral perfusion and oxygenation as well as drainage of cerebral spinal fluid (CSF) and brain interstitial fluid waste products (Louveau et al., 2015). Increased age is a known risk factor for cerebrovascular dysfunction and dementia (Alzheimer’s Association, 2014). In addition, there are a number of pathophysiological conditions that can increase cardiovascular risk and thus increase the risk for changes in cerebral blood flow including heart disease, hypertension, atherosclerosis and diabetes (see Hays et al., 2018).

Cellular mechanisms responsible for the regulation of cerebral blood flow, such as endothelial nitric oxide (NO) availability may also be involved in age-related changes in brain health (Katusic and Austin, 2014). A recent study using arterial spin labeled perfusion MRI (Venturelli et al., 2018) showed that healthy older participants showed reductions in both cerebral blood flow and NO bioavailability compared to younger individuals, and those with MCI and AD showed further declines. Both changes were correlated with age-related declines in cognitive function, suggesting that changes in NO and cerebral blood flow may be related, and important for cognitive health.

Brain health drivers related to brain vasculature include not only cerebral blood vasculature and the delivery of oxygen to the brain but also the brain lymphatic vascular systems involved in CSF turnover and waste removal. The discovery of the CNS meningeal lymphatic vascular system (Aspelund et al., 2015; Louveau et al., 2015) has opened new areas of understanding of how the age-related changes in the brain lymphatic circulation may affect brain health. A recent pre-clinical study of young and old mice demonstrated that there is a significant age-dependent decline in CSF recirculation *via* the paravascular lymphatic system (Kress et al., 2014). This dysfunction in the older mice was ameliorated when the meningeal lymphatic circulation was augmented with meningeal transfection with vascular endothelial growth factor C. While these studies have yet to be confirmed in humans, together they suggest that the meningeal lymphatic vascular system is also an important component of healthy brain aging.

The blood-brain-barrier (BBB), comprised of cerebrovascular endothelial cells and perivascular mural cells and pericytes, serves to protect the brain from circulating neurotoxins and pathogens. Age-related degeneration of the BBB is linked to neuropathologies which is an important risk category for ARCI. Studies from brain autopsies in individuals diagnosed with AD have demonstrated damage to the BBB and infiltration of blood-related proteins in the hippocampus and cortex Post-mortem studies have shown BBB damage in AD including accumulation in the hippocampus and cortex of blood-derived proteins (Baloyannis and Baloyannis, 2012; Sengillo et al., 2013). Recent studies in the living human brain using contrast MRI have shown age-related decreases in BBB integrity in the region of the hippocampus (Montagne et al., 2015). These studies compared BBB integrity between individuals with MCI and age-matched cognitively normal individuals. They reported greater BBB breakdown in the MCI group suggesting that decreased BBB integrity may be a driver of ARCI.

### Neuropathologies

Neuropathologic changes in the aging brain often include changes in extracellular amyloid-β (Aβ) in plaques, increases in intracellular hyperphosphorylated tau and neurofibrillary tangles (NFTs), with α-synuclein, TDP-43 proteinopathy, and hippocampal sclerosis occurring less frequently (Rahimi and Kovacs, 2014). These neuropathological changes have been suggested to drive alterations in functional synaptic circuits and neuron loss. While some of the ARCI risk categories, such as immune dysfunction and cardiovascular insufficiency are linked to increased neuropathologies, other risk factors are not. For example, glucose dysregulation including Type 1 and 2 diabetes, which is a key risk category associated with ARCI, has not been found to be associated with increases in neuritic plaques and neurofibrillary tangles identified with an AD diagnosis (Biessels and Despa, 2018). Some of these pathologies, such Aβ plaques and tangles, develop in relatively predictable patterns, while others, such as cerebrovascular pathology, can be highly variable in terms of type, cause, location, and consequence (O’Brien and Thomas, 2015).

Postmortem neuropathological (Driscoll et al., 2006; O’Brien et al., 2009) and biomarker studies (e.g., Toledo et al., 2015; Vemuri and Knopman, 2016), as well as recent studies using positron emission tomography (PET; Aizenstein et al., 2008; Resnick et al., 2010; Resnick and Sojkova, 2011) indicate that approximately 30% of cognitively normal individuals have some level of increased Aβ plaques. In individuals without dementia, this neuropathology has been shown to decrease cognition cross-sectionally and to increase the rate of cognitive decline (e.g., Vemuri et al., 2015; Duke Han et al., 2017).

In normal functioning neurons, tau is a prominent microtubule-associated protein involved in the assembly of tubulin into microtubules and structure stabilization (Weingarten et al., 1975). In AD, tau protein is hyperphosphorylated and results in NFT that correlate quite well with the degree of cognitive impairment in AD (Cho et al., 2016; Schöll et al., 2016). The impact of NFTs on normal aging is still being defined (Crary et al., 2014). The term primary age related tauopathy (PART) has been used to describe a continuum of NFT distribution from focally-distributed in cognitively normal individuals to tau-predominant in dementia. Generally, PART occurs with minimal to absent β-amyloid pathology and may account for as much as 18% of pathologies in cognitively normal older individuals (Knopman et al., 2003; Josephs et al., 2017).

A third protein, alpha-synuclein, is also important to consider, along with Aβ and hyperphosphorylated tau. Abnormal aggregation of alpha-synuclein is involved in Parkinson’s disease and Lewy Body dementia as well as in synaptic dysfunction (Colom-Cadena et al., 2013). A study of plasma levels in healthy older males showed a significant decrease in alpha-synuclein between the 3rd and 5th decade of life (Koehler et al., 2015), indicating that these changes may reflect normal age-related changes in protein homeostasis.

The combination of AD, cerebrovascular, and Lewy body pathologies account for significant variance in longitudinal rates of global cognitive decline among older adults (Boyle et al., 2013; Power et al., 2018). However, even after accounting for the presence of these pathologies, the majority of variance in cognitive decline remains unexplained. Additionally, there are a number of reports in which individuals show substantial neuropathology, such as Aβ plaques (Jack and Holtzman, 2013) without cognitive decline. Together, these findings raise the possibility that there may be “resilience factors” that protect some individuals from developing cognitive impairment in the presence of these neuropathologies (Dickson, 1997; Murray and Dickson, 2014)—possibly including genomics, lifetime experiences, measures related to neuronal integrity and synaptic function, or individual differences in neurotransmitter system functions (e.g., Kaasinen et al., 2000; Barulli and Stern, 2013). More research is needed to understand whether the effects of multiple pathologies are independent or synergistic, and how they are modified by other risk or resilience factors, such as overall health, genetic predisposition, and lifestyle factors.

### Neuron and Synaptic Function

Each of the drivers of brain health discussed above—inflammation, blood flow, neuropathologies and their associated risk categories—ultimately interact to affect neuron function. Neurons do not work in isolation. Rather, they function as interconnected networks within and across brain regions. The neural circuit dynamics that support cognition can be affected by cerebral blood vascularization and oxygen delivery, brain lymphatic vascular efficiency, by toxic Aβ, tau or alpha-synuclein species, or by changes in the function of glia-neuron interactions. In addition, neuron to neuron communication through the synapse is altered by both normative and pathological aging processes, directly impacting network function (Eastwood et al., 2006; Burke and Barnes, 2010; Schimanski et al., 2013). Critically, functional synapses are necessary for enabling the computations required to support high levels of cognition.

Data from humans, nonhuman primates and rodents all suggest relative preservation of neuron number and morphology in normative aging (Burke and Barnes, 2010). It has also been demonstrated that neuronal membrane dynamics and other biophysical properties of old cells are well preserved with age (Rosenzweig and Barnes, 2003) in animal models of aging. Synapse number and function, however, are clearly impacted by age across species. The structure and function of aging synapses have been examined most extensively in two primary regions of the brain—the hippocampus and the prefrontal cortex. Both of these regions play important, independent roles in cognition, and these structures are highly interconnected.

The number of synaptic contacts made onto hippocampus and prefrontal cortex cells in the rodent and nonhuman primate declines with age, but these changes appear to be restricted to certain synaptic input types, spine types and regional cell types. For example, in the rodent hippocampus, synaptic loss is selective to synapses from layer II medial entorhinal projections to granule cells and CA3 pyramidal cells (Geinisman et al., 1992; Smith et al., 2000). In the nonhuman primate prefrontal cortex, the reduction in synaptic contacts occurs specifically in those synapses made onto thin dendritic spines (Morrison and Baxter, 2012).

While the exact triggers responsible for loss of anatomical and functional synapses across the lifespan are not completely understood, alterations in gene transcription and translation are clearly involved (Fraga et al., 2005; Starnawska et al., 2017; Barter and Foster, 2018). For example, there are *increases* with age in genes related to neuroinflammation, oxidative stress, mitochondrial and calcium dyshomeostasis (Prolla, 2002; Blalock et al., 2003; Ianov et al., 2016, 2017a). In addition, there is *reduced* expression of hippocampal and prefrontal cortical genes linked to synaptic structure and plasticity in the aging brain, as well as changes in expression of immediate early genes (IEGs) that are responsive to neuron activity (e.g., Prolla, 2002; Blalock et al., 2003; Penner et al., 2011; Ianov et al., 2016, 2017a; Barter and Foster, 2018). In neurons of both regions, epigenetic increases in methylation of synaptic genes appear to result in decreased transcription (Penner et al., 2011, 2016; Ianov et al., 2016, 2017b). Critically, these changes in synaptic genes are linked to cognitive impairment in aged animals (Penner et al., 2011; Ianov et al., 2017b). Thus, the co-occurrence of an increase in genes involved in neuroinflammation (e.g., Mangold et al., 2017) with a decrease in synaptic genes suggest that brain or systemic inflammation may be an important driver of changes at the aging synapse.

Not only are synapses lost in aging, but the synaptic plasticity mechanisms thought to reflect the biological basis of stable memories are also altered in the aging process (e.g., Barnes, 1979). In the rodent, both long-term potentiation (LTP) that strengthens synaptic communication, and long-term depression (LTD) that weakens synaptic strength are altered in aging (Deupree et al., 1993; Norris et al., 1996; Rosenzweig and Barnes, 2003). For example, the durability of LTP is correlated with spatial memory in individual rats (e.g., Barnes, 1979)—i.e., the better the memory the more durable LTP is over weeks. Old spatial memory-impaired rats have the fastest decaying LTP and LTP is harder to induce at old hippocampal synapses than in young. The age-related changes in plasticity mechanisms may be partly explained by dysregulation of microRNAs that influence translation of synaptic genes into proteins (Siegel et al., 2011; Danka Mohammed et al., 2017) that affect LTP (Ryan et al., 2017) and LTD (Fiore et al., 2014) mechanisms in hippocampus.

Among the processes that underlie synapse health and synaptic plasticity is the activation of genes in the IEG family. For example, the IEG Arc (activity-regulated cytoskeleton-associated protein; Lyford et al., 1995) is important both for spatial memory consolidation and the persistence of hippocampal LTP (Guzowski et al., 2000; Plath et al., 2006), and provides an excellent single cell marker of behavior-induced circuit activity (Guzowski et al., 1999). Penner et al. (2011) showed that behaviorally-induced transcription of *Arc* is altered in aging, and this may in part be due to a higher level of methylation of the Arc gene that restricts transcription.

A second IEG that is known to be important for memory stabilization is Neuronal Pentraxin 2 (NPTX2), also known as Narp (Tsui et al., 1996; Chang et al., 2010). NPTX2 is thought to maintain the excitatory-inhibitory balance within brain circuits, and we know that this balance is disrupted in aging, from single cell recording and immunohistochemistry experiments in rats (Wilson I. A. et al., 2005; Spiegel et al., 2013) and monkeys (Thome et al., 2012), and from MRI experiments in humans (Yassa et al., 2011). Recent studies in humans have shown a significant reduction in NPTX2 in CSF of AD patients (Swanson and Willette, 2016; Xiao et al., 2017) that correlate with hippocampal volume and cognitive function. Additionally, data support the idea that high levels of NPTX2 can serve as a “resilience factor” to protect brain circuit function and cognition, even in those with significant neuropathology (Xiao et al., 2017).

The changes in synaptic connections and synaptic plasticity that occur with age predict that network function should also be altered in aging. Spatial navigation and memory depend on the function of the hippocampus (O’Keefe and Nadel, 1978), and are altered in aging across all species investigated—including mice, rats, dogs, monkeys and humans (Lester et al., 2017). Two different kinds of circuit dysfunction have been observed in old, spatial memory-impaired rats. One is an age-related change in CA1 pyramidal cells that involves the “CA1 place field map” becoming unstable in old rats. Even in familiar environments, sometimes old rats’ CA1 place fields “remap” (change their spatial distribution) when they should not. In CA3, on the other hand, old rats’ “CA3 place field map” becomes rigid, and sometimes does not remap when it should, even from one distinctly different environment to another.

In summary, enabling neuron and synaptic health is key to preserving cognitive health in aging. Each of the other brain drivers of ARCI can impact synaptic and circuit function *via* multiple pathways. Understanding individual differences in the mechanisms through which synapses and circuits are changed with age will be fundamental to implementing Precision Aging approaches to maintain brain and cognitive health across the lifespan.

## Biomarkers Related to Brain Drivers

Our model suggests that the primary targets for intervention to improve the cognitive life-span are the brain drivers that lead to cognitive impairments. Thus, a critical companion to cognitive testing is an ability to ascertain the molecular state of the brain as well as an individual’s inherited genetic factors associated with ARCI. Circulating biomarkers—molecules that can be isolated from biological fluids and/or cells in the blood—will play an important role in understanding an individual’s risk for ARCI because such molecules exhibit changes before such effects are evident through standardized cognitive testing. For example, the cerebrospinal levels of amyloid-β fragments are known to decrease years before a diagnosis of AD is reached clinically (Skoog et al., 2003; Moonis et al., 2005; Gustafson et al., 2007; Stomrud et al., 2007). Whether the same basic concept applies to ARCI is unknown, however. Are there molecules that can serve as early indicators of ARCI? Do these molecules change before, after, or during the point at which the alteration can be recognized through cognitive testing? What role can these markers play in determining an individual’s risk/protective profile for ARCI? The development of a panel of molecular tests that are informative for each class of brain driver, including neural inflammation, blood flow, neuropathology, and neuron function, is key for creating individualized panels of risk as well as determining the impact of therapeutic interventions. Examples of known biomarkers related to the four classes of brain drivers are discussed briefly below.

### Biomarkers of Inflammation

Markers of brain inflammation are of interest to characterize in the context of cognitive aging due to the known role of immune and inflammatory processes on brain health and disease. It is important to note that peripheral and central biomarkers of inflammation can be different and therefore the source of the biospecimen (CSF vs. blood) must be considered as well. Also of interest is the comparison between peripheral inflammation biomarkers and central inflammatory biomarkers as predictors of cognition. Very little is known about this because it requires the collection of blood and CSF from each study participant.

The three most studied biomarkers of inflammation and cognition are IL-6, TNF alpha, and C-reactive protein. In fact, these molecules are closely connected in a signaling cascade whereby IL-6 stimulates TNF-alpha production and CRP levels rise as a consequence of the resulting inflammation. Multiple lines of research have suggested that these cytokines are altered in both ARCI and dementia. A recent meta-analysis (Bradburn et al., 2018) demonstrated that levels of IL-6—a pro-inflammatory cytokine that can cross the blood brain barrier—in plasma can be predictive of cognitive decline over a 2–7 year follow-up period such that those with higher circulating levels of IL-6 were at increased risk for cognitive decline. TNF-alpha, a pro-inflammatory cytokine produced primarily by activated macrophages but also neurons, was found to be elevated with aging in general in a study of centenarians, and higher levels were associated with dementia (Bruunsgaard et al., 1999). C-reactive protein was found to be elevated in non-demented participants of the Northern Manhattan Study who demonstrated higher risk for impaired memory (Noble et al., 2010). The understanding of the molecular inflammatory profile—both circulating and central—is critical to fully characterize an individual’s inflammation status in the context of cognitive aging.

### Biomarkers of Neuropathology

Biomarkers of neurodegenerative brain disorders including Alzheimer’s (AD) and Parkinson’s (PD) have been well characterized from the aspect of CSF, and to a lesser extent, blood-based assessments. Currently amyloid-β fragments (Aβ1–40 and 1–42), total tau, and phosphorylated tau levels are of interest in the CSF of individuals with suspected, or at high risk for, AD. The ratios of these biomarkers can be utilized to aid in diagnostic accuracy in suspected AD. Importantly, they can predict progression from mild cognitive impairment or even cognitively normal status to AD up to 10 years in advance (Rosenmann, 2012). Recent findings in the field of extracellular vesicles provide promise for CNS-specific biomarkers of neuropathology and neurodegenerative disease (Chen et al., 2017). In the CNS, neurons as well as astrocytes, microglia, and oligodendrocytes secrete exosomes into the extracellular space. Exosomes act as mediators delivering important proteins, short interfering RNA (siRNA), and microRNAs (miRNAs) that aid in intercellular communication (De Toro et al., 2015). Alterations in microRNA profiles in exosomes associated with pathologies such as beta and tau are measurable in CSF and blood, providing promise for new biomarkers in the diagnosis of AD (Cheng et al., 2015).

### Biomarkers of Neuron Function

An ability to assess neuron function using a molecular biomarker approach would be of significant utility in understanding an individual’s brain physiology. Recent work using CSF-based measurements of the NPTX2 and GluA4 proteins show promise as neural function molecular biomarkers. NPTX2 is a presynaptically-expressed immediate-early gene whose expression can regulate the levels of the AMPA receptor subunit, GluA4, in PV-positive interneurons. By assaying these markers in the CSF one can begin to assess the circuit dynamics of the brain in a quantitative fashion. Higher CSF levels of NPTX2 have been correlated with less MTL atrophy and cognitive decline across 2 years (Swanson and Willette, 2016). NPTX2 and GluA4 levels are reduced in the cortex of AD patients and CSF measurements of NPTX2 show reductions as well, and both levels correlate with cognitive function and hippocampal volume (Xiao et al., 2017). One critical next step for the assay of these biomarkers would be to measure them in the blood as this would vastly improve the ease with which they could be studied in larger aging cohorts. Biomarkers related to this brain driver are of particular interest because they may serve as a molecular phenotype of neural circuit function that may precede the actual functional changes such as the development of ARCI or cognitive decline.

Our model suggests that the presence of risk categories will alter specific brain drivers which, in turn, will result in a predicted biomarker signal for that altered brain driver. However, there are likely examples where biomarker signals may remain within the normal range, even in the presence of significant risk. This could be due to our inability to measure the brain driver biomarker signal with sufficient sensitivity. Alternatively, this could represent a particular resistance in an individual to brain driver changes, which is also of great interest. Identifying and adding well validated and reliable biomarkers for brain drivers can only be addressed through the in-depth molecular and phenotypic study of large longitudinal cohorts of healthy older adults.

## The Precision Aging Model in Practice

**Figure 3:**
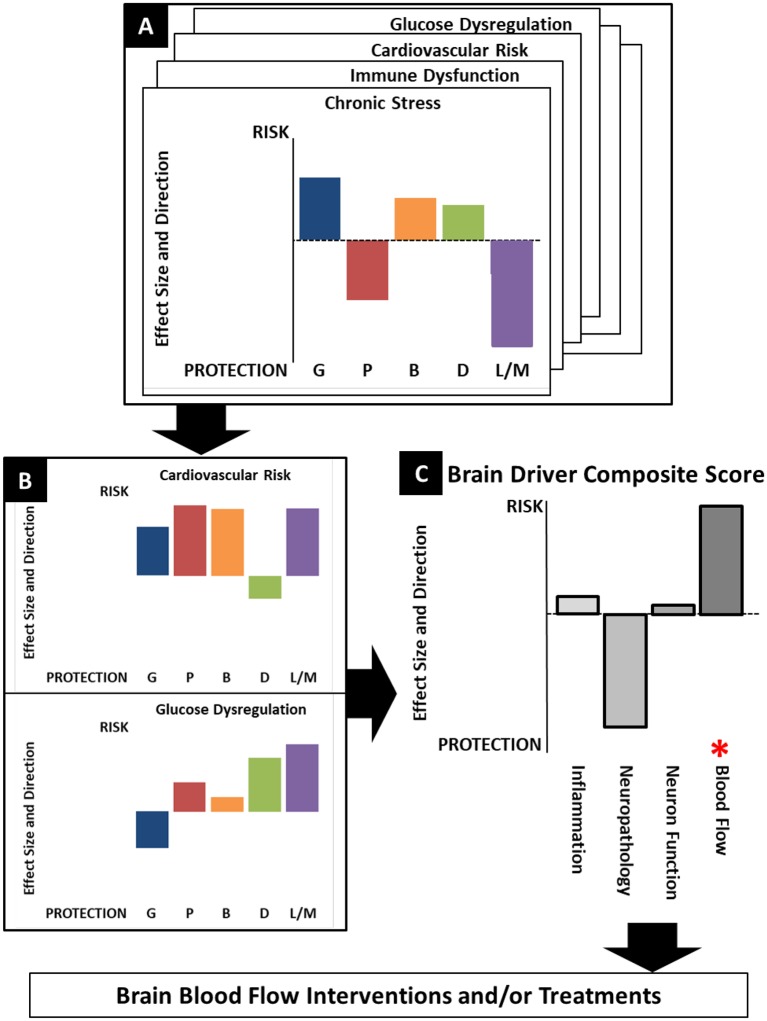
A conceptualization of the Precision Aging model in practice, showing the workflow from assessments of individual risk categories **(A)** to identifying significant risks **(B)** which are combined in a composite score that reflects risk for each brain driver. **(C)** Both the brain driver composite scores and the contributing risk categories lead to the choice of optimal treatments to ameliorate risk factors and to address the at-risk brain driver directly.

Figure 3 provides a conceptual representation of the Precision Aging approach in practice as it might apply to a given individual. Panel A represents detailed assessments of each of the previously described risk categories (e.g., Chronic Stress, Immune Dysfunction, Cardiovascular Risk, Glucose Dysregulation). Each of the categories will include assessments of multiple measurable classes of variables that may include genetics (G), phenotype (P), Biomarkers (B), Demographics (D), and Lifestyle/Medical factors (L/M). The specific factors contributing to a particular variable class will differ depending on the risk category, with the combination and weighting of factors derived empirically. Each variable class for a risk category is illustrated by the colored bars, with differing bar heights indicating the degree of risk association or protection. As a concrete example, in Figure 3A, this individual shows a moderately high genetic risk for chronic stress (Genetic—G). However, their genetic risk may be offset by positive lifestyle factors including a fulfilling job, an excellent social support system, and no reported sleep disturbance (Lifestyle/Medical—L/M). As a result, while they show mildly elevated markers of inflammation (Biomarkers—B), they report overall high levels of life satisfaction and low levels of daily stress (Phenotype—P). Despite the genetic risk, then, this individual may have an overall chronic stress score that is at or below the midpoint between risk and protection.

Elevated risk category scores can then be considered in combination with one another (Figure 3B) to derive brain driver composite scores (Figure 3C). Note that some risk category scores may be relevant to more than one Brain Driver. For example, cardiovascular risk may contribute to both the Inflammation and Blood Flow composite scores, while chronic stress may contribute most heavily to the Inflammation brain driver. In the example illustrated here, this individual has strongly elevated risk for cardiovascular risk (a family history of heart disease, uncontrolled hypertension, poor diet and low physical activity) coupled with a moderately increased risk for glucose dysregulation (obesity, high resting glucose levels), making this individual at highest risk for the Blood Flow brain driver.

The information presented in this fashion has several advantages. First, it allows an individual to easily digest a summary of complex information that will highlight areas of highest concern. The chart should be used to guide the user and their primary care physician towards interventions that improve a profile by targeting underlying risk factor contributions as well as more specific interventions targeted to specific Brain Drivers. This latter type of intervention will become more feasible as direct biomarkers of brain drivers become more available. Second, the chart system would provide the individual with a way to dynamically explore outcomes. For example, as an individual incorporates lifestyle changes that move them towards normal body weight or bring their blood pressure to within normal limits, they could see the immediate impact of these changes on their risk category scores and the downstream impact they would have on Brain Driver scores. Lifestyle changes are notoriously difficult to implement and even more difficult to sustain, and such positive feedback may be both motivating and empowering for individuals.

## Moving the Field Forward

In the previous sections, we discussed some of the areas of research that will be necessary to address if we are to make progress toward our stated goal of maintaining brain health across the full extent of the adult lifespan. In order to put the Precision Aging model into practice, we need a better understanding of how risk factors relate to one another to form categories of risk, how multiple specific factors accumulate risk within a given risk category, and which categories of risk account for the greatest amount of variance in ARCI and cognitive decline. For most categories of risk, we do not yet have a clear understanding of how they are linked to brain drivers that set the stage for accelerated age-related brain injury and cognitive dysfunction. Additionally, risk categories must be combined with personalized genetic, lifestyle and demographic profiles to create individualized intervention strategies that combine preventative and therapeutic approaches. In order to evaluate the molecular state of the brain and assess the impact of interventions, there is a critical need for novel biomarkers that reflect specific brain drivers known to be associated with ARCI. Ideally, these biomarkers would be easily attainable (i.e., through circulating blood) and inexpensive so that they can be used in large scale studies of cognitive aging and become accessible as a tool for primary medical care. Several additional important points regarding a research agenda are briefly discussed below.

### Big Data and the Complexity of Aging

Clearly, the aging process is incredibly complicated. To capture that complexity, large data sets are required that include multiple layers of evaluation, both cross sectional and longitudinal, in order to identify categories of interrelated risk factors, how risk factors are moderated by genes, and how they interact with demographic factors including sex, race, socioeconomic status, geographic location, education, and others. Big data sets also require analytic methods for reducing complexity by identifying those combinations of factors that account for the largest amounts of variance in trajectories of cognitive aging, and those factors that are most predictive of future cognitive decline. Ideally, such data sets would include measurements obtained in the context of an individual’s daily life while they engage in tasks that are meaningful, by using the internet, mobile devices, and sensors to capture cognition, activity, sleep, and other aspects of functional capacity. One way to address the enormous scale of data required for such an enterprise is to share information across large-scale longitudinal studies and existing biobanks that are funded through federal agencies.

The ultimate goal of big data analytic methods is to reduce complexity to its simplest form. In the end, instead of requiring the assessment of hundreds of different risk factors, creating a profile of brain health may require only a handful of key predictors that will lead to effective interventions to prevent or ameliorate ARCI. If that is the case, a pared-down version of the Precision Aging model could be readily implemented on a smart phone or web-based assessment for use by a primary care physician or an individual. That endpoint is likely only achievable, however, through the analysis of large-scale, multifaceted and complex data sets.

### The Impact of Interventions on Brain Drivers

One important implication of the Precision Aging model is that interventions, to be most effective, should ameliorate the impact of brain drivers, rather than solely focusing on reducing or removing a specific risk factor. However, just as we do not have a clear understanding of the link between risk categories and brain drivers, we have a limited understanding of the mechanisms by which interventions impact the brain. As discussed earlier, interventions that may not appear similar on the surface may, in fact, provide the same benefit to the brain because they share a common underlying mechanism. Understanding treatments from this perspective—specifically, their impact on brain drivers—will allow us to tailor the ideal intervention (or combination of preventative and therapeutic interventions) to an individual based not only on their profile of risk but also on their own preferences. One person may find meditation boring and difficult to practice, but they may enjoy engaging in meaningful social interaction such as volunteering for a local school, both of which may have the same long-term impact on brain health by decreasing chronic stress. A treatment regimen is only as good as a person’s willingness to adhere to it.

The ideal treatment regimen should combine preventative interventions that focus directly on decreasing specific risk factors with therapeutic interventions that ameliorate the brain injury that has already taken place. For example, a smoking cessation program is important to prevent continued exposure to noxious chemicals, while pharmacological interventions may be required to reverse the damage that has already occurred. Additionally, providing individualized interventions in the home, rather than solely in the clinic, and providing people with continuous feedback and follow-up, will be key to ensuring that interventions will be effective and reach a sufficiently large segment of the population.

## Summary

Our hope is that the Precision Aging model can lead to novel advances in the measurement, prevention, and treatment of ARCI by creating individualized risk profiles that are linked to a customized intervention plan. Ultimately, our approach could lead to a *diagnostic system* that enables primary health care providers to identify and implement precision solutions for sustaining cognitive health. By working to match cognitive health with lifespan, such a system would decrease hospitalization time, extend independent living, improve productivity and quality of life, and decrease the risk for AD.

## Author Contributions

LR was responsible for the overview, model, and future directions section of the article. BL, TR, CP, AS and MM contributed to the risk categories subsections of the article. MH and CB wrote the section on brain drivers. MJH contributed the section on genetics and biomarkers of brain function. All authors contributed to the development of the model and provided revisions of drafts.

## Conflict of Interest Statement

The authors declare that the research was conducted in the absence of any commercial or financial relationships that could be construed as a potential conflict of interest. The handling editor declared a past collaboration with one of the authors CB.
